# Digital Surveillance: A Novel Approach to Monitoring the Illegal Wildlife Trade

**DOI:** 10.1371/journal.pone.0051156

**Published:** 2012-12-07

**Authors:** Amy L. Sonricker Hansen, Annie Li, Damien Joly, Sumiko Mekaru, John S. Brownstein

**Affiliations:** 1 HealthMap, Children’s Hospital Informatics Program, Harvard–Massachusetts Institute of Technology Division of Health Sciences and Technology, Boston, Massachusetts, United States of America; 2 Division of Emergency Medicine, Children’s Hospital Boston, Boston, Massachusetts, United States of America; 3 State University of New York at Albany, School of Public Health, Department of Epidemiology and Biostatistics, Rensselaer, New York, United States of America; 4 City University of Hong Kong, Department of Biology and Chemistry, Kowloon, Hong Kong; 5 Wildlife Health Program, Wildlife Conservation Society, Nanaimo, British Columbia, Canada; 6 Boston University, School of Public Health, Department of Epidemiology, Boston, Massachusetts, United States of America; 7 Departments of Medicine and Epidemiology, Biostatistics and Occupational Health, McGill University, Montreal, Quebec, Canada; 8 Department of Pediatrics, Harvard Medical School, Boston, Massachusetts, United States of America; USGS National Wildlife Health Center, United States of America

## Abstract

A dearth of information obscures the true scale of the global illegal trade in wildlife. Herein, we introduce an automated web crawling surveillance system developed to monitor reports on illegally traded wildlife. A resource for enforcement officials as well as the general public, the freely available website, http://www.healthmap.org/wildlifetrade, provides a customizable visualization of worldwide reports on interceptions of illegally traded wildlife and wildlife products. From August 1, 2010 to July 31, 2011, publicly available English language illegal wildlife trade reports from official and unofficial sources were collected and categorized by location and species involved. During this interval, 858 illegal wildlife trade reports were collected from 89 countries. Countries with the highest number of reports included India (n = 146, 15.6%), the United States (n = 143, 15.3%), South Africa (n = 75, 8.0%), China (n = 41, 4.4%), and Vietnam (n = 37, 4.0%). Species reported as traded or poached included elephants (n = 107, 12.5%), rhinoceros (n = 103, 12.0%), tigers (n = 68, 7.9%), leopards (n = 54, 6.3%), and pangolins (n = 45, 5.2%). The use of unofficial data sources, such as online news sites and social networks, to collect information on international wildlife trade augments traditional approaches drawing on official reporting and presents a novel source of intelligence with which to monitor and collect news in support of enforcement against this threat to wildlife conservation worldwide.

## Introduction

The true worth of the illegal wildlife trade is unknown. This multi-faceted and clandestine industry has disrupted fragile ecosystems and facilitated the spread of pathogens and novel infectious diseases in humans, domestic animals, and native wildlife [1], [2]. The trade includes live and dead wildlife of multiple species that are captured, poached, and sold for food, medicine, pets and trophies [3]. While some data exist on the volume, scope and scale of the global wildlife trade, the current understanding of the network is largely inferred from data on legal import and exports recorded by the Convention on International Trade in Endangered Species of Wild Fauna and Flora (CITES) treaty, which requires member nations to document global trade in endangered wildlife [4]. The vast illegal trade remains largely unmonitored and underground.

Not only is the true global scale of the illegal wildlife trade unknown, but also regional and local levels of wildlife trade are difficult to assess [5]. Quantifying all global wildlife trade would be a herculean task, as illegal trade routes range in scale from local to international levels and are often conducted through informal networks [2]. Confiscation records provide much of the only available data on the scope of this illegal network. A 2010 study conducted by Rosen and Smith assessed the scope and scale of the worldwide illegal wildlife trade by examining 12 years of seizure records compiled by TRAFFIC, an international wildlife trade-monitoring network. The study found 967 documented seizures of illegal wildlife and wildlife products representing vast species diversity and geographic scope. Factors such as inadequate infrastructure, corrupt officials, international crime networks, and a shortage of environmental conservation law enforcement officers affected individual nation’s seizure activity [6].

Previous attempts to monitor the illegal wildlife trade have had mixed results. In 2002, the Invasive Species Internet Monitoring System was implemented to track Internet trade of invasive species using a semi-automated process of searching for these species sold online. Refining the search parameters within semi-automated queries allowed the system to monitor Internet sales of CITES listed species. While the system provided excellent results supporting proof of concept, it only captured Internet sales, which may not be representative of the greater illegal trade picture. In addition, the query parameters had to be well-defined for each species of interest in order to obtain relevant search results, and query results had to be consistently reviewed by subject matter experts in order to determine if further action needed to be taken [7]. The sacrifice of sensitivity for specificity combined with its labor-intensive approach limited its scalability.

A second study employed wildlife trade market surveys, which were administered repeatedly to more accurately estimate the number of illegally traded animals [8]. While repeat market surveys may improve the estimation of a particular species being illegally traded in specific regions or localities, obtaining a more accurate estimate of internationally illegally traded wildlife would require a much more robust system built to survey a multitude of markets worldwide for a wide array of species [5].

Illegal wildlife trade can be intercepted and reported by both official (e.g. government agencies, non-profit organizations working with local officials) and unofficial sources (e.g. news media). A surveillance system that collects both unofficial and official data may offer a more complete picture of the trade by providing up-to-date, highly localized information on the illegal commercialization of wildlife, providing a picture of the illegal wildlife trade that until now has not been visualized on a global scale. To the best of our knowledge, an automated, real-time, comprehensive, global system monitoring official and unofficial reports of illegal wildlife trade activity has not previously been put into practice. Such a system would undoubtedly be useful to wildlife authorities in tracking illegal wildlife trade. Herein, we introduce an automated digital surveillance system that was developed to monitor reports on illegally traded wildlife and wildlife products.

## Materials and Methods

### Website Development

An automated digital surveillance system was developed that utilizes natural language processing and machine-learning algorithms to combine unofficial and official reports of wildlife trade events obtained from the Internet in an effort to establish an automated web crawling surveillance system of the wildlife trade, similar to those used for infectious disease events (e.g. GPHIN, HealthMap) [9], [10]. The underlying architecture of the HealthMap system has been previously described [11]. Briefly, the system addresses the challenge of scouring the Internet for pertinent outbreak information through automated querying, filtering, and visualization of reports through the utilization of automated text processing algorithms that classify alerts by location and disease [10]. This flexible system has been adapted for use in other non-disease surveillance settings [12].

**Figure 1:**
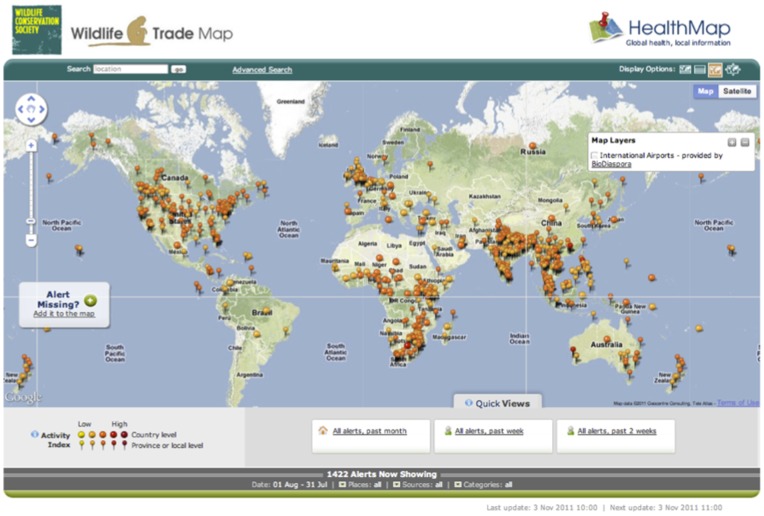
Wildlife trade website showing time period from August 1, 2010 to July 31, 2011. Illegal wildlife trade reports received through the automated system are shown with the orange pins. Screenshot taken November 3, 2011. www.healthmap.org/wildlifetrade.

Available at http://www.healthmap.org/wildlifetrade, the digital wildlife surveillance tool is freely available and displays real-time reports of illegal wildlife trade activity worldwide as an interactive visualization (Figure 1). The system collects reports based on the keyword search strings described below and utilizes text-mining algorithms to classify reports on the illegal wildlife trade by location and species prior to overlaying this information onto an interactive mapping tool. Reports were gathered from around the world but were limited to English for this proof-of-concept development. The resulting system continually aggregates, organizes, and disseminates near real-time information to provide insight into the global wildlife trade network. We focus our analysis on one year of illegal wildlife and wildlife product confiscation data collected between August 1, 2010 and July 31, 2011.

### Report Sources and Detection

Valuable information about the illegal wildlife trade is available via the Internet through official and unofficial sources. Official sources from freely available RSS feeds were utilized and included TRAFFIC, WildAid, The Coalition Against Wildlife Trafficking (CAWT), World Wildlife Fund (WWF), and the International Fund for Animal Welfare (IFAW). In addition, reports from unofficial sources were automatically collected utilizing key search terms from freely available websites, discussion forums, mailing lists, news media outlets, and blogs. Information was obtained only from publically available sources to respect privacy issues. Standard Internet citation was practiced by including a brief excerpt from the original article and then linking to the source for additional detail. Overall, the system capitalizes on news indexers that draw from over 50,000 possible web-based resources [13].

### Keyword Selection

The development and utilization of key search terms allows information on the global wildlife trade network from disparate unofficial sources to be monitored in near real-time. Upon reviewing historical reports on the illegal wildlife trade, we created a small set of candidate keywords that were both relevant and fairly specific to the illegal wildlife trade. We tested the potential of those keywords as queries to retrieve reports within the Google Reader tool (which allows the retrieval of a Google News query as an RSS feed). New keywords were added as determined by browsing for relevant reports that our system was missing. These new keywords were used to increase the amount of results yielded or to improve the specificity of the query. In order to collect reports most relevant to the illegal wildlife trade, there were several rounds of query improvements.

As an example, the word “poaching” would often appear, and was therefore used as an initial keyword. However, used alone, the term brought in reports unrelated to the wildlife trade. We added inclusion and exclusion terms to narrow the focus such as “in title: poaching wildlife” and “in title: poaching –bank –egg.” This process was conducted repeatedly, building on our initial set of candidate keywords, to obtain relevant and specific information on the wildlife trade. A total of 18 search terms were selected to gather the reports analyzed here.

### Data Collection and Visualization

**Figure 2:**
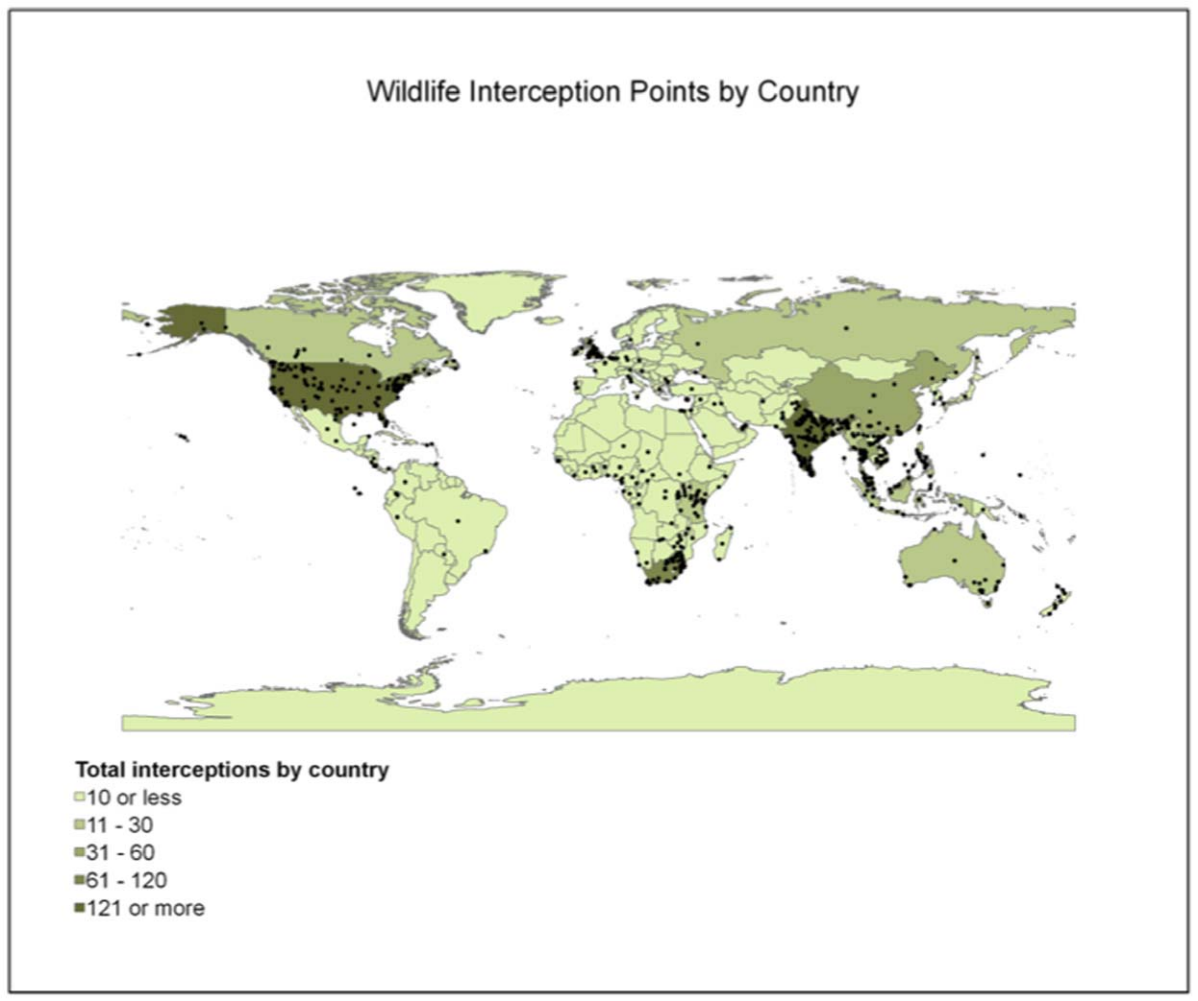
Illegal wildlife trade interception points with darker areas showing countries with greater numbers of wildlife and wildlife products intercepted by enforcement officials. ESRI 2011. ArcGIS Desktop: Release 10.

For each report yielded by the selected keywords, the system extracted specific details including the species involved in the report, the specific geographical location where interception of illegal wildlife product occurred (Figure 2), a link to the original information source, and the date of source publication. Due to reporting inconsistencies (e.g. time of occurrence was sometimes reported as ongoing or as specific as a year, month, or week), the time of illegal wildlife trade activity was not included in statistical analysis.

Each report collected was reviewed manually by an analyst to ensure the most accurate information was displayed on the website. The analyst added precise geographical details for interception location and species information as needed. In addition, the analyst ensured that duplicate reports were hidden from display and that reports were correctly tagged. Category tags were applied to improve filtering [11], and include *Breaking*, for articles pertaining to live animal or wildlife product seizures with specific information on species, date, and location; *Warning*, for articles with details about historically known illegal wildlife trade activity, articles describing increasing levels of illegal trade over a period of time, or regarding illegal wildlife trade routes commonly utilized; and *Context*, for alerts on policy, law, or collaborations involving the illegal wildlife trade without specific incident information on product seizures or trade routes used. Reports tagged as *Breaking* or *Warning* appear on the wildlife trade website, while reports tagged as *Context* do not appear on the site to minimize information overload. Our analysis focused on *Breaking* alerts only.

To avoid confusion, commonly traded wildlife species were displayed on the website as shown in Table 1, broken down into common names and broad categories instead of listed by scientific names. When possible, detailed scientific names of species were recorded in an internal database in order to determine each species’ red list status (the conservation status determined by the International Union for Conservation of Nature). Transportation methods used were also recorded when information was available.

**Table 1:** Species categories developed for the HealthMap Wildlife Trade website.

Species Classification				
Level 1	Level 2	Level 3	Level 4	Level 5
Amphibians				
Amphibians	Frogs			
Amphibians	Newts			
Amphibians	Salamanders			
Amphibians	Toads			
Aquatic Species				
Aquatic Species	Coral			
Aquatic Species	Elasmonbrachii			
Aquatic Species	Elasmonbrachii	Rays		
Aquatic Species	Elasmonbrachii	Sharks		
Aquatic Species	Elasmonbrachii	Skates		
Aquatic Species	Fish (osteichthyes)			
Aquatic Species	Fish (osteichthyes)	Eel		
Aquatic Species	Fish (osteichthyes)	Seahorse		
Aquatic Species	Fish (osteichthyes)	Sturgeon		
Aquatic Species	Marine Mammals			
Aquatic Species	Marine Mammals	Cetecea		
Aquatic Species	Marine Mammals	Cetecea	Dolphins	
Aquatic Species	Marine Mammals	Cetecea	Porpoise	
Aquatic Species	Marine Mammals	Cetecea	Whales	
Aquatic Species	Marine Mammals	Cetecea	Whales	Beluga
Aquatic Species	Marine Mammals	Cetecea	Whales	Pilot
Aquatic Species	Marine Mammals	Cetecea	Whales	Right
Aquatic Species	Marine Mammals	Pinnipeds		
Aquatic Species	Marine Mammals	Pinnipeds	Sea Lions	
Aquatic Species	Marine Mammals	Pinnipeds	Seals	
Aquatic Species	Marine Mammals	Pinnipeds	Walrus	
Aquatic Species	Marine Mammals	Sirenia		
Aquatic Species	Marine Mammals	Sirenia	Dugong	
Aquatic Species	Marine Mammals	Sirenia	Manatee	
Aquatic Species	Shellfish			
Aquatic Species	Shellfish	Crustaceans		
Aquatic Species	Shellfish	Crustaceans	Crabs	
Aquatic Species	Shellfish	Crustaceans	Lobsters	
Aquatic Species	Shellfish	Crustaceans	Shrimp	
Aquatic Species	Shellfish	Echinoderms		
Aquatic Species	Shellfish	Echinoderms	Sea Cucumber	
Aquatic Species	Shellfish	Echinoderms	Sea Urchins	
Aquatic Species	Shellfish	Echinoderms	Starfish	
Aquatic Species	Shellfish	Molluscs		
Aquatic Species	Shellfish	Molluscs	Abalone	
Aquatic Species	Shellfish	Molluscs	Clams	
Aquatic Species	Shellfish	Molluscs	Mussels	
Aquatic Species	Shellfish	Molluscs	Octopus	
Aquatic Species	Shellfish	Molluscs	Oysters	
Aquatic Species	Shellfish	Molluscs	Paua	
Aquatic Species	Shellfish	Molluscs	Squid	
Arthropods				
Arthropods	Insects			
Arthropods	Spiders			
Arthropods	Spiders	Tarantulas		
Birds				
Birds	Accipitriformes (Birds of Prey)			
Birds	Accipitriformes (Birds of Prey)	Eagles		
Birds	Accipitriformes (Birds of Prey)	Eagles	Bald Eagles	
Birds	Accipitriformes (Birds of Prey)	Eagles	Golden Eagles	
Birds	Accipitriformes (Birds of Prey)	Hawks		
Birds	Accipitriformes (Birds of Prey)	Vultures		
Birds	Anseriformes			
Birds	Anseriformes	Ducks (domestic)		
Birds	Anseriformes	Ducks (wild)		
Birds	Anseriformes	Geese		
Birds	Anseriformes	Swan		
Birds	Columbiformes			
Birds	Columbiformes	Doves		
Birds	Columbiformes	Pigeons		
Birds	Falconiformes			
Birds	Falconiformes	Falcons		
Birds	Galliformes			
Birds	Galliformes	Chicken		
Birds	Galliformes	Peacocks		
Birds	Galliformes	Pheasant (domestic)		
Birds	Galliformes	Pheasant (wild)		
Birds	Galliformes	Poultry (domestic)		
Birds	Galliformes	Quail		
Birds	Galliformes	Turkey		
Birds	Passerines (song birds)			
Birds	Passerines (song birds)	Crows		
Birds	Pelicaniformes			
Birds	Pelicaniformes	Pelican		
Birds	Psittaciformes			
Birds	Psittaciformes	Parakeet		
Birds	Psittaciformes	Parrots		
Birds	Ratites			
Birds	Ratites	Emus		
Birds	Ratites	Ostrich		
Birds	Sphenisciformes			
Birds	Sphenisciformes	Penguins		
Birds	Strigiformes			
Birds	Strigiformes	Owls		
Mammals				
Mammals	Anteaters			
Mammals	Armadillo			
Mammals	Bats			
Mammals	Bears			
Mammals	Cats (wild)			
Mammals	Cats (wild)	Bobcats		
Mammals	Cats (wild)	Cheetah		
Mammals	Cats (wild)	Civets		
Mammals	Cats (wild)	Jaguars		
Mammals	Cats (wild)	Leopards		
Mammals	Cats (wild)	Lions		
Mammals	Cats (wild)	Lynx		
Mammals	Cats (wild)	Mountain lions		
Mammals	Cats (wild)	Panthers		
Mammals	Cats (wild)	Puma		
Mammals	Cats (wild)	Tigers		
Mammals	Dogs (wild)			
Mammals	Dogs (wild)	Coyote		
Mammals	Dogs (wild)	Fox		
Mammals	Dogs (wild)	Hyena		
Mammals	Dogs (wild)	Jackal		
Mammals	Dogs (wild)	Wolf		
Mammals	Elephant			
Mammals	Hoofstock			
Mammals	Hoofstock	Antelope		
Mammals	Hoofstock	Boar		
Mammals	Hoofstock	Buffalo (wild)		
Mammals	Hoofstock	Deer		
Mammals	Hoofstock	Elk		
Mammals	Hoofstock	Giraffes		
Mammals	Hoofstock	Hippopotamus		
Mammals	Hoofstock	Moose (wild)		
Mammals	Hoofstock	Rhinoceros		
Mammals	Hoofstock	Sheep (wild)		
Mammals	Hoofstock	Springbok		
Mammals	Hoofstock	Tapirs		
Mammals	Hoofstock	Zebra		
Mammals	Lagomorphs			
Mammals	Lagomorphs	Pikas		
Mammals	Lagomorphs	Rabbits (wild)		
Mammals	Livestock (domestic)			
Mammals	Livestock (domestic)	Alpaca		
Mammals	Livestock (domestic)	Buffalo		
Mammals	Livestock (domestic)	Camel		
Mammals	Livestock (domestic)	Cows		
Mammals	Livestock (domestic)	Goats		
Mammals	Livestock (domestic)	Horses		
Mammals	Livestock (domestic)	Llama		
Mammals	Livestock (domestic)	Pigs		
Mammals	Livestock (domestic)	Sheep		
Mammals	Marsupials			
Mammals	Marsupials	Kangaroos		
Mammals	Marsupials	Koala		
Mammals	Marsupials	Sugar Gliders		
Mammals	Marsupials	Tasmanian Devil		
Mammals	Marsupials	Wombat		
Mammals	Mole			
Mammals	Mongoose			
Mammals	Opossum			
Mammals	Panda			
Mammals	Pangolins			
Mammals	Pets (domestic)			
Mammals	Pets (domestic)	Cats		
Mammals	Pets (domestic)	Chinchilla		
Mammals	Pets (domestic)	Dogs		
Mammals	Pets (domestic)	Ferrets		
Mammals	Pets (domestic)	Guinea pig		
Mammals	Pets (domestic)	Hamster/gerbil		
Mammals	Pets (domestic)	Hedgehog		
Mammals	Pets (domestic)	Mice		
Mammals	Pets (domestic)	Rabbits		
Mammals	Pets (domestic)	Rats		
Mammals	Primates			
Mammals	Primates	Apes		
Mammals	Primates	Apes	Chimpanzees	
Mammals	Primates	Apes	Gibbon	
Mammals	Primates	Apes	Gorillas	
Mammals	Primates	Apes	Orangutans	
Mammals	Primates	Baboon		
Mammals	Primates	Lemurs		
Mammals	Primates	Loris		
Mammals	Primates	Monkeys		
Mammals	Raccoon			
Mammals	Rodents			
Mammals	Rodents	Beaver		
Mammals	Rodents	Chipmunk		
Mammals	Rodents	Gambian Pouched Rat		
Mammals	Rodents	Mouse		
Mammals	Rodents	Porcupine		
Mammals	Rodents	Prairie Dog		
Mammals	Rodents	Rat		
Mammals	Rodents	Squirrel		
Mammals	Skunks			
Mammals	Sloth			
Mammals	Weasel family			
Mammals	Weasel family	Badger		
Mammals	Weasel family	Mink		
Mammals	Weasel family	Otter		
Mammals	Weasel family	Weasel		
Mammals	Weasel family	Wolverine		
Non-human animal				
Reptiles				
Reptiles	Alligator			
Reptiles	Crocodile			
Reptiles	Lizards			
Reptiles	Lizards	Gecko		
Reptiles	Lizards	Gecko	Jeweled	
Reptiles	Lizards	Gecko	Tokay	
Reptiles	Snakes			
Reptiles	Snakes	Cobras		
Reptiles	Snakes	Pythons		
Reptiles	Turtles			
Reptiles	Turtles	Sea Turtles		
Reptiles	Turtles	Terrapins/Freshwater Turtles		
Reptiles	Turtles	Tortoise		

The HealthMap architecture allows for flexible categorization of species, therefore phyla, classes and orders were only used when they provided a useful categorical container. For example, “Marine Mammals” is useful when an article does not provide more specific species information. On the other hand, skunks are not listed as “Mammals - Carnivora – Skunks” because an intermediate category of Carnivora has not been needed. The species categories can be easily updated, and additional intermediate or more specific groupings are added as needed.

Locations of wildlife interception points were visually displayed on the website along with a link to the original information source. Last, a geographical layer featuring international airports was added, as previous studies have highlighted airports as being important points of entry of illegal wildlife products [14]–[16]. For example, one study estimated that five tons of bushmeat was smuggled per week into the Paris Roissy-Charles de Gaulle airport alone [14]. Showing airports may allow officials to identify major transportation hubs for the illegal wildlife trade.

### Comparison with CITES

To confirm the underlying assumption that the volume of wildlife traded illegally is not accurately reflected through the legal trade of wildlife products, we examined the CITES database of legally traded, threatened or potentially threatened species. The CITES database is “based on a system whereby permits or certificates are issued for international trade in specimens of species listed in one of three Appendices, each of which provides a different degree of trade control [17].” CITES data on gross imports of elephants (both the *Elephas* and *Loxodonta* genera), which were the most commonly intercepted based on our findings, were analyzed for 2008, 2009, and 2010. Data from 2011 was not yet available.

## Results

From August 1, 2010 to July 31, 2011, 858 reports of illegal wildlife trade were collected for analysis. Reports on illegal wildlife crime were reported from a total of 89 countries. Countries with the highest number of reports on wildlife trade included India (n = 146, 15.6%), the United States (n = 143, 15.3%), South Africa (n = 75, 8.0%), China (n = 41, 4.4%), and Vietnam (n = 37, 4.0%). Species from 118 categories were recorded. The species traded or poached were recorded, with mammals being the most commonly reported. Specifically, elephants (n = 107, 12.5%), rhinoceros (n = 103, 12.0%), tigers (n = 68, 7.9%), leopards (n = 54, 6.3%), and pangolins (n = 45, 5.2%) were the most commonly intercepted mammals. Reasons for wildlife product trade ranged vastly (Table 2).

**Table 2:** Common reasons for the illegal trade of wildlife and wildlife products.

Major Category	Sub-category	Wildlife Product Examples
Medicinal		
	Aphrodisiac	Tiger penis soaked in brandy or made into soups
	Traditional	Rhinoceros horn to treat fever, cure cancer and alleviate other ailments
		Tiger bone made into tonics to treat joint problems and lessen pain
		Bear bile for inflammation and infection
		Pangolin scales to reduce swelling
		Manta ray gills to remove toxins
	Quackery	Sea turtle eggs as a cure for asthma
		Tokay gecko as a cure for HIV
Pet Trade		
	Exotic	Tropical fish
		Tiger cubs and other large cats
		Slow loris
	Collector	Ball pythons, chameleons, and other species as part of reptile trade
	Live Export/Other	Dolphins for marine parks and aquariums
		Owls for children in India following popular Harry Potter book series
Sport		
	Traditional	Falcon eggs for field sport of falconry
Human Consumption		
	Delicacy	Shark fins for Asian soup
		Beluga sturgeon roe as highly coveted black caviar
	Sustenance	Monkeys and chimpanzee species consumed as bushmeat and source of protein in developing countries
	Vitality	Tokay gecko wine or whiskey to increase strength and energy
Ornamental		
	Trophy	Stuffed birds of prey (hawks, owls, and eagles) for display
	Souvenirs	Elephant ivory carved into chopsticks
	Jewelry	Red coral used to make earrings and other fashion accessories
		Hair clips made from shells of the nearly extinct green sea turtle
	Home Goods	Sperm whale teeth with etchings and engravings
	Art	Butterflies for artistic greeting cards, paper weights, and other decorative products
	Clothing	Red panda fur for coats and hats
		Shatoosh scarves woven from hair of Tibetan antelope, which must be killed in order to obtain hair
Religious		
	Ritual	Sea turtles sacrificed as part of Hindu ritual
		Leopard pelts worn during ceremonies conducted by followers of Shembe religion
		Bald and golden eagle parts and feathers for Native American religious ceremonies
	Superstition	Critically endangered radiated tortoise thought to bring good luck if owned
		Owls often sacrificed on auspicious occasions
	Black Magic/Sorcery	Body parts of owls are valued by black magic and sorcery practitioners
		Leopard nails utilized for tantric/black magic practices

CITES data showed that *Elephas* genera imports averaged 13,661 from 2008–2010, and were comprised of both live animals and animal products ranging from ivory carvings to meat and hair. The *Loxodonta* genera averaged 89,523 from 2008–2010 comprised of live animals in addition to products such as bone carvings, ears, feet, hair, ivory carvings, meat, skins, skulls, and teeth [18]. It should be noted that according to CITES, gross import data is often an overestimation of the quantity actually traded, as where different quantities have been reported by the importer and the exporter, the program selects the larger of the two quantities [17].

## Discussion

The HealthMap Wildlife Trade website is a comprehensive digital surveillance system for aggregating, organizing, and displaying illegal wildlife trade reports from official and unofficial sources. By providing a unified platform of both official and unofficial information, users are able to quickly and easily view a plethora of information (whether by species or location) from one application. From August 1, 2010 to July 31, 2011, 858 reports of illegal wildlife trade were collected from 89 countries illustrating the global extent of this lucrative industry.

Previous methods that have attempted to estimate the enormity of the illegal wildlife trade have focused primarily on traditional data sets of legally traded wildlife. However, substantial amounts of information may be obtained through the utilization of unofficial digital media sources, as demonstrated by the number of reports collected, highlighting just one of the benefits of considering this methodology. Additionally, the data collected may prove useful to both conservation and public health officials by providing real-time and detailed information on whereabouts of threatened species and species capable of transmitting zoonotic diseases.

There are several other organizations that apply a variety of methods for monitoring illegal wildlife trade activity, but due to differences in scope and purpose, direct comparisons are difficult. The WWF, in partnership with TRAFFIC, created the Law Enforcement Management Information System (LEMIS) tracker that plots official data and flows of wildlife products seized upon entry into the United States (http://wildlifetradetracker.org/). As compared to LEMIS, the HealthMap wildlife trade system covers wildlife products seized worldwide and includes unofficial sources of information. It also allows users to view original media reports on seizures made, where LEMIS does not provide access to source information. The HealthMap wildlife trade map does not show wildlife product flows at this time.

The Tiger Tracker, also created by WWF and TRAFFIC, plots official data on seizures of tigers and tiger parts within Asia. HealthMap’s wildlife trade site covers the illegal trade for all species of wildlife worldwide. For 2010, the Tiger Tracker showed 13 tiger product seizures from 7 countries (Nepal, India, Thailand, Malaysia, Vietnam, Indonesia, and China). Using the advanced search tool on the HealthMap wildlife trade map, breaking news alerts for 2010 were searched specifically for the same 7 countries previously listed, and 17 alerts involving tigers were obtained. It is difficult to compare the alerts however, as the Tiger Tracker does not consistently provide links to the original information sources.

Other organizations such as Save the Elephants, Freeland, Lusaka Agreement Task Force, Wildlife Direct, ASEAN-WEN Wildlife Enforcement Network, Wildlife Alliance, and Interpol compile alerts on the illegal wildlife trade. Compared to these systems, HealthMap’s wildlife trade monitoring site is more specific, showing alerts that explicitly involve seizures of live animals or of wildlife products. In addition, the HealthMap wildlife trade site allows users to sort alerts by species seized, location, and date. While other sites show all articles on the wildlife trade, HealthMap focuses on illegal wildlife seizures, hiding articles on policy, collaboration between countries to fight wildlife trafficking, or articles on seizures involving plant or tree materials to prevent information overload.

Lastly, the use of submissions from the general public is an important feature that distinguishes our system from most others. Citizen science data has been shown to have the potential to detect and track disease events and trends earlier than official data sources [19], [20]. While Freeland allows the general public to contribute information on suspected wildlife trafficking in Southeast Asia, it likely receives a different subset of information from the public. Their website states, “we will act,” suggesting that all publically submitted reports are thoroughly investigated and therefore it is likely to elicit eyewitness reports of illegal trade rather than the web-available news articles, press releases, and government statements on the wildlife trade events that our users report. Submissions to the HealthMap wildlife trade site are later reviewed by trained staff to check for duplicates, and to ensure removal of any personally identifying information. If a submitted alert warrants further investigation, our protocol is to refer it to collaborators at WCS.

The International Union for the Conservation of Nature (IUCN) provides a list of species at risk of extinction called the Red List of Threatened Species. Threatened species may be poached or caught live and sold for a high value because of their novelty in the market. The system detected reports involving numerous species of concern including the near-threatened pangolin for its scales, the critically endangered black rhino for its horn, the endangered Javan slow loris for the exotic pet trade, the vulnerable mandrill for bushmeat, and many others [21]. The presence of IUCN Red List species in our digital surveillance underscores the need to prevent illegal trade.

When compared to the CITES database, illegal wildlife trade reports collected through our website included 107, or 12.5%, reports involving illegal interceptions of elephant products. However, just one interception in our database may involve many products from many different species. As an example, one report collected from December 2010 involved the confiscation of 105 pieces of jewelry made of elephant ivory [22], while another 2 tons of elephant ivory, or 247 tusks, were intercepted in April 2011 [23]. This would therefore make these two reported interceptions of elephant products equivalent to 352 products if listed in the CITES database, as the CITES database counts each product individually (i.e. an elephant carving, hide, or skull). It is difficult to make a direct comparison to the CITES data due to the difference in wildlife product categorizations, however further research is being conducted to obtain a better understanding of the scope of the illegal wildlife trade.

Surveillance of illegal wildlife trade activity may also bring insight into the spread of zoonotic diseases as an early detection system, as the trade has been shown to play an inarguable role in the facilitation of disease transmission [24]. Jones et al. found that a majority of emerging infectious diseases were caused by zoonotic pathogens, and that over 70% originated in wildlife, with the number of events increasing significantly over time [25]. The wildlife trade contributes to the potential for increasing numbers of emerging diseases, as humans are directly exposed to wildlife and wildlife products through the many varying aspects of this lucrative industry (e.g. via hunters, salesmen, consumers) [2]. History has shown the dangers of zoonotic disease transmission through commercialization of live wildlife and their products [16], [26]. Examples of past outbreaks from wildlife trade include monkeypox, which was imported into the United States in 2003 when infected Gambian pouched rats (Cricetomys gambianus) were transported with pet prairie dogs (Cynomys ludovicianus) [27]. Other cases of zoonotic diseases have also occurred and include rabies from mammals, severe acute respiratory syndrome (SARS) from small carnivores, highly pathogenic avian influenza from avian species, and chytridiomycosis from amphibians [2], [28], [29]. In light of these threats the USAID’s Emerging Pandemic Threats Program: Predict Project strives to build a global early warning system for emerging diseases that are transmitted between wildlife and people.

Surveillance of illegal trafficking is inherently limited by the clandestine nature of the activity; underreporting is unavoidable. Additional biases may occur by selection of key search terms, media reporting biases, and restriction to the English language. For instance, a wealthy country may have more resources (such as Internet access, higher regulation standards, and freedom of press) than a less economically developed country. This lack of resources may prevent the detection and reporting of illegally traded wildlife despite the possibility of more trade occurring in underdeveloped areas.

The search for illegally traded items is also limited by the key word search terms utilized. Although a sample of queries was repeatedly tested to select for the most appropriate search terms, selection bias may have still resulted. There may also be additional biases towards certain terms selected or species reportedly traded. Only events reported through media outlets, or announced through official channels in which RSS feeds are being followed (CAWT, IFAW, TRAFFIC, WildAid, and the WWF), are shown on the website. Therefore, a bias exists towards stories that appeal to each media outlet’s target audience and stories deemed to be particularly “newsworthy”, such as those concerning particularly large seizures or that focus on charismatic megafauna like elephants, rhinoceros, and tigers. To the degree that media biases result in underreporting of lesser-known species that are important to the eco-system at large, this system will suffer from mirrored underreporting.

The system does attempt to capture reports regardless of species involved through its utilization of specific key word search terms. Terms like “seizure” or “illegal wildlife trade” are not synonymous with specific species. If our key search terms are actually used more often in stories for certain species of animals, then those animals may be better represented than others.

Lastly, media reports may not accurately reflect the true legality of each event reported. It may later be determined during legal proceedings that a transport of a wildlife product reported as illegal was actually being legally conducted. For products involving endangered species however, where any transport or sale is prohibited, the number of reports incorrectly categorized is likely minimal.

Further, using only one language (English) may have caused an over-reporting in English speaking countries, such as India (n = 146, 15.6%), the United States (n = 143, 15.3%), and South Africa (n = 75, 8.0%). Previous literature has shown Asia to be a focal point of illegal wildlife trafficking, and these past findings were more in line with the next highest-ranking countries, China (n = 41, 4.4%) and Vietnam (n = 37, 4.0%) where English is not the main language [30]. Our preliminary results show that it would be beneficial to include non-English language reports from additional RSS feeds to obtain a more complete picture of the illegal wildlife trade. We have therefore begun to monitor reports in Japanese and plan to add Chinese, Malay, and Indonesian languages next. Adjustment for English-language Internet news sites per country was not possible under the scope of this pilot study.

Additionally, it would be useful to look into biases of species considered when conducting surveillance of illegal wildlife trade. For instance, in the United States deer and elk were often confiscated but not typically discussed by wildlife trade organizations due to their current status of “not threatened.” The higher activity level within the United States in our automated system may have been due to the inclusion of deer, elk, bear, and moose being illegally poached or traded. Monitoring the trade in species not currently classified as threatened or endangered should be considered, as they may be at heightened risk for future threat. In addition, zoonotic diseases may be transmitted regardless of a species’ conservation status.

Future work may also concentrate on analysis of media outlets, funding for enforcement, and user demographics. In addition, work may be conducted to display wildlife trade routes to and from a location, transportation methods used, and wildlife red list status of species confiscated. Additional geographical layers such as animal densities and transportation hubs may aid in further understanding the illegal wildlife trade and its associations with geographical factors. Additional work linking species traded with zoonotic diseases may also be conducted to identify potential hot-spot regions for emerging zoonotic diseases.

Last, methods used to transport wildlife were documented when available, but not included in our results due to the low proportion of reports providing transportation information (19%). Insight into transportation methods could aid regulators in intercepting illegal wildlife trade before it reaches its final destination, and therefore as the dataset grows we hope to identify useful patterns. From the subset of reports that did include transportation methods of illegal wildlife trade (n = 163), modes of transportation utilized varied greatly and included land vehicles (trucks, buses, and cars), airplanes, boats, and trains. Some also utilized the Internet to purchase wildlife products that were then shipped through the mail (via ground and air postal services). The use of the Internet as a resource for obtaining illegal wildlife products is not new, however as Internet access becomes more readily available this may be an area to monitor closely. The wildlife trade website may aid in highlighting trends such as the utilization of the Internet for illegal wildlife trafficking in addition to shedding light on interceptions in areas not typically emphasized.

Despite the limitations to a digital surveillance system, the HealthMap Wildlife Trade website is currently the most comprehensive and freely available tool for monitoring the illegal wildlife trade and may help improve our understanding of a clandestine market. The illegal wildlife trade continues to grow, and new challenges are consistently emerging, such as an increased number of online sales of illegal wildlife with limited regulation [31], [32]. The problem continues to receive limited resources and political attention, as it escapes detection through an underground economy [33]. Further, this illegal industry is worsened by urbanization and global development that commercializes subsistence hunting and fishing and over-exploits terrestrial and marine ecosystems [34]. To keep up with these advances, global digital surveillance of the illegal wildlife trade is necessary to protect biodiversity, prevent endangerment of species, and control the introduction of infectious diseases [35], [36].

## References

[1] Gratwicke B, Evans MJ, Jenkins PT, Kusrini MD, Moore RD, et al (2010) Is the international frog legs trade a potential vector for deadly amphibian pathogens? Front Ecol Environ 8: 438–442.

[2] Karesh WB, Cook RA, Bennett EL, Newcomb J (2005) Wildlife trade and global disease emergence. Emerg Infect Dis 11: 1000–1002. 16022772 10.3201/eid1107.050194 PMC3371803

[3] TRAFFIC International (2008) Our Work: wildlife trade. Available: http://www.traffic.org/trade/. Accessed: 2012 May 29.

[4] World Wildlife Fund (1994) How CITES works. In: Hemley G, editor. International Wildlife Trade: A CITES Sourcebook. Washington, D.C.: Island Press. 1–9.

[5] Barber-Meyer SM (2010) Dealing with the clandestine nature of wildlife-trade market surveys. Conserv Biol 24: 918–923. 20408867 10.1111/j.1523-1739.2010.01500.x

[6] Rosen GE, Smith KF (2010) Summarizing the evidence on the international trade in illegal wildlife. Ecohealth 7: 24–32. 20524140 10.1007/s10393-010-0317-y PMC7087942

[7] Suiter K, Sferrazza S. Monitoring the sale and trafficking of invasive vertebrate species using automated internet search and surveillance tools. In: Witmer GW, Pitt WC, Fagerstone KA, editors; 2007; USDA/APHIS/WS, National Wildlife Research Center, Fort Collins, CO. 90–93.

[8] TRAFFIC Southeast Asia (2007) Tiger trade revisited in Sumatra, Indonesia. Petaling Jaya, Malaysia. Available: http://www.worldwildlife.org/species/finder/tigers/WWFBinaryitem9544.pdf. Accessed: 2012 Aug 13.

[9] Brownstein JS, Freifeld CC, Reis BY, Mandl KD (2008) Surveillance Sans Frontieres: Internet-based emerging infectious disease intelligence and the HealthMap project. PLoS Med 5: e151. 18613747 10.1371/journal.pmed.0050151 PMC2443186

[10] Freifeld CC, Mandl KD, Reis BY, Brownstein JS (2008) HealthMap: global infectious disease monitoring through automated classification and visualization of Internet media reports. J Am Med Inform Assoc 15: 150–157. 18096908 10.1197/jamia.M2544 PMC2274789

[11] Brownstein JS, Freifeld CC (2007) HealthMap: the development of automated real-time internet surveillance for epidemic intelligence. Euro Surveill 12: E071129 071125. 10.2807/esw.12.48.03322-en 18053570

[12] Giles J (2012) System tracks violence in Syrian uprising. New Scientist 213: 22.

[13] Google News (2012) About Google News. Available: http://support.google.com/news/bin/answer.py?hl=en&answer=106259&topic=2428790&ctx=topic. Accessed: 2012 July 31.

[14] Chaber A-L, Allebone-Webb S, Lignereux Y, Cunningham AA, Marcus Rowcliffe J (2010) The scale of illegal meat importation from Africa to Europe via Paris. Conservation Letters 3: 317–321.

[15] Warchol GL (2004) The Transnational Illegal Wildlife Trade. Criminal Justice Studies 17: 57–73.

[16] Smith KM, Anthony SJ, Switzer WM, Epstein JH, Seimon T, et al (2012) Zoonotic viruses associated with illegally imported wildlife products. PLoS One 7: e29505. 22253731 10.1371/journal.pone.0029505 PMC3254615

[17] CITES (2010) A guide to using the CITES Trade Database. Available: http://www.unep-wcmc-apps.org/citestrade/docs/CITESTradeDatabaseGuide_v7.pdf. Accessed: 2012 Jan 16.

[18] CITES trade statistics derived from the CITES Trade Database, UNEP World Conservation Monitoring Centre, Cambridge, UK.

[19] Freifeld CC, Chunara R, Mekaru SR, Chan EH, Kass-Hout T, et al (2010) Participatory epidemiology: use of mobile phones for community-based health reporting. PLoS Med 7: e1000376. 21151888 10.1371/journal.pmed.1000376 PMC2998443

[20] Dickinson JL, Zuckerberg B, Bonter DN (2010) Citizen Science as an Ecological Research Tool: Challenges and Benefits. Annual Review of Ecology, Evolution, and Systematics 41: 149–172.

[21] International Union for Conservation of Nature (2011) The IUCN Red List for Threatened Species. Available: http://www.iucnredlist.org. Accessed: 2011 Nov 18.

[22] (2010) Kenya arrests Thai woman in airport ivory bust. InDepth Africa Magazine. Available: http://indepthafrica.com/news/east-africa/kenya-arrests-thai-woman-in-airport-ivory-bust/. Accessed: 2010 December 26.

[23] (2011) Thais seize 2 tons of ivory in largest bust ever. King Broadcasting Company. Available: http://www.kgw.com/news/world/119047354.html. Accessed: 2011 Apr 1.

[24] Travis DA, Watson RP, Tauer A (2011) The spread of pathogens through trade in wildlife. Rev Sci Tech 30: 219–239. 21809766 10.20506/rst.30.1.2035

[25] Jones KE, Patel NG, Levy MA, Storeygard A, Balk D, et al (2008) Global trends in emerging infectious diseases. Nature 451: 990–993. 18288193 10.1038/nature06536 PMC5960580

[26] Marano N, Arguin PM, Pappaioanou M (2007) Impact of globalization and animal trade on infectious disease ecology. Emerg Infect Dis 13: 1807–1809. 18258027 10.3201/eid1312.071276 PMC2876780

[27] Guarner J, Johnson BJ, Paddock CD, Shieh WJ, Goldsmith CS, et al (2004) Monkeypox transmission and pathogenesis in prairie dogs. Emerg Infect Dis 10: 426–431. 15109408 10.3201/eid1003.030878 PMC3322777

[28] Bell D, Roberton S, Hunter PR (2004) Animal origins of SARS coronavirus: possible links with the international trade in small carnivores. Philos Trans R Soc Lond B Biol Sci 359: 1107–1114. 15306396 10.1098/rstb.2004.1492 PMC1693393

[29] Weldon C, du Preez LH, Hyatt AD, Muller R, Spears R (2004) Origin of the amphibian chytrid fungus. Emerg Infect Dis 10: 2100–2105. 15663845 10.3201/eid1012.030804 PMC3323396

[30] Nijman V (2010) An overview of international wildlife trade from Southeast Asia. Biodivers Conserv 19: 1101–1114.

[31] Wu J (2007) World Without Borders: Wildlife trade on the Chinese-language Internet. TRAFFIC Bulletin 21: 75–84.

[32] International Fund for Animal Welfare (2008) Killing With Keystrokes: Wildlife trade on the Internet. London. Available: http://www.ifaw.org/united-states/node/900. Accessed: 2011 Oct 31.

[33] Wellsmith M (2011) Wildlife Crime: The Problems of Enforcement. European Journal on Criminal Policy and Research 17: 125–148.

[34] Baker CS (2008) A truer measure of the market: the molecular ecology of fisheries and wildlife trade. Mol Ecol 17: 3985–3998. 18643915 10.1111/j.1365-294X.2008.03867.x

[35] Childs JE, Mackenzie JS, Richt JA (2007) Wildlife and Emerging Zoonotic Diseases: The biology, circumstances and consequences of cross-species transmission. Curr Top Microbiol Immunol 315: 477–508.17848076

[36] Smith KF, Behrens M, Schloegel LM, Marano N, Burgiel S, et al (2009) Ecology. Reducing the risks of the wildlife trade. Science 324: 594–595. 19407185 10.1126/science.1174460

